# Aspartic protease 2 from *Trichinella spiralis* excretion/secretion products hydrolyzes tight junctions of intestinal epithelial cells

**DOI:** 10.1371/journal.pntd.0013805

**Published:** 2025-12-08

**Authors:** Shao Rong Long, Hui Ran Zhang, Jing Jing Wang, Zi Xuan Liao, Qi Xue Fan, Yu Qing Liang, Cheng Yu Gan, Ruo Dan Liu, Jing Cui, Xi Zhang, Zhong Quan Wang, Xin Qi

**Affiliations:** 1 Department of Pathogen Biology, Zhengzhou University School of Basic Medical Sciences, Zhengzhou, China; 2 Department of Blood Transfusion, The Affiliated Cancer Hospital of Zhengzhou University & Henan Cancer Hospital, Zhengzhou, China; Instituto de Salud Carlos III, SPAIN

## Abstract

*Trichinella spiralis* is a globally distributed foodborne parasitic nematode causing zoonotic infections. The invasion and subsequent development of intestinal infective larvae within the intestinal mucosa are critical steps in *T. spiralis* infection of the host. Previous studies have demonstrated that aspartic protease 2 from *Trichinella spiralis* excretion/secretion products (TsASP2) plays a role in facilitating the invasion of host intestinal epithelial cells by the larvae; however, the underlying mechanism remains unclear. Studies have suggested that dysfunction of the intestinal barrier creates a more favorable environment for intestinal helminths to invade intestinal epithelial cells. Here, the role of recombinant TsASP2 (rTsASP2) in disrupting the intestinal barrier by hydrolyzing tight junctions (TJs) was analyzed using both *in vitro* and *in vivo* experiments. A *Lactococcus lactis*- based heterologous protein delivery system was constructed to deliver TsASP2 to intestinal mucosa. The expression and mucosal colonization of rTsASP2 in *L. lactis,* the integrity of the intestinal epithelium, and the degradation of TJ proteins and hydrolysis sites of rTsASP2 were confirmed with Western blot, immunofluorescence, transepithelial electrical resistance (TEER) detection, paracellular permeability to FITC-dextran, transmission electron microscopy (TEM), dextran sulfate sodium (DSS)-induced colitis model, and High-Performance Liquid Chromatography Coupled to Time-of-Flight Mass Spectrometry (HPLC-TOF/MS). Results revealed TsASP2 delivery system using *L. lactis* was successfully construction and capable of colonizing the gut and delivering rTsASP2 to the intestinal mucosa. The rTsASP2 could directly degrade the TJ proteins Occludin and Claudin-1, thereby increasing paracellular permeability in the intestinal barrier and exacerbating DSS- induced colitis in mice. HPLC-TOF/MS analysis of the bands generated by rTsASP2- mediated hydrolysis of TJ protein specifically identified the cleavage sites as K-S, R-A, R-T, R-E and R-L. These results elucidated the mechanism by which TsASP2 facilitates *T. spiralis* invasion through the hydrolysis of tight junction proteins, providing critical experimental evidence for understanding host-parasite interactions.

## Author summary

Humans acquire trichinellosis by consuming raw pork infected with *T. spiralis* muscle larvae. A critical step in establishing infection is the larva’s breach of the intestinal barrier to continue development. Between intestinal epithelial cells, tight junctions (TJs) act as “gate-like structures” that seal the intestinal barrier. Studies have showed that excretion/secretion products (ESPs) of *T. spiralis* exert a regulatory influence on the junctional proteins of intestinal epithelial cells. However, whether proteases from ESPs directly hydrolyze the junctional proteins of intestinal epithelial cells remains unclear. Here, we demonstrate that *T. spiralis* aspartic protease 2 (TsASP2), derived from the ESPs of *T. spiralis*, can hydrolyze TJ proteins, thereby compromising their barrier function and increasing intestinal permeability. This study provides the evidence that *T. spiralis* ESPs facilitate larval invasion by direct degradation of TJs. Furthermore, a heterologous mucosal protein delivery system was developed using *Lactococcus lactis*. By delivering TsASP2 to the host intestinal mucosa, this system effectively mimics the functional role of ESPs during *T. spiralis* invasion, offering a valuable reference approach for investigating the molecular mechanisms of helminth invasion.

## Introduction

As a globally significant foodborne zoonotic pathogen, *Trichinella* has been reported to infect more than 100 mammalian species [1]. There are about 10, 000 recorded cases of human *Trichinella* infection per year in the world [2]. A total of 6,662 cases of human trichinellosis were confirmed in Argentina between 2012 and 2018, and 258 cases were confirmed in Chile between 2005 and 2015 [3]. The European Food Safety Authority and the European Centre for Disease Prevention and Control reported 398 confirmed cases of human trichinellosis across 27 European Union countries from 2018 to 2022 [4]. In China, eight outbreaks of human trichinellosis occurred between 2009 and 2020, resulting in 479 cases and 2 deaths [5].

Humans contract *Trichinella* by consuming raw or uncooked meat that carries infective muscle larvae [6,7]. Upon entering the digestive system, the meat is degraded in the stomach, liberating larvae from their protective collagen capsules. The bile and intestinal contents play a crucial role in mediating the infective behavior of certain intestinal helminths, including *Heligmosomoides polygyrus* and *Trichinella spiralis*. It is well documented that the infective larvae of *Heligmosomoides polygyrus* actively penetrate the duodenal intestinal wall, with bile serving as a key stimulus for this process. Notably, experimental studies have demonstrated that when the bile duct is surgically redirected to the ileum, larval penetration shifts accordingly to the ileal region- precisely at the site of bile delivery [8]. Further support for the role of intestinal factors comes from in vitro studies on *Trichinella spiralis*: larvae isolated from rat intestines or incubated with intestinal contents exhibit significantly enhanced invasive capacity compared to those maintained in saline alone [9]. Subsequent exposure to bile and intestinal contents within the gastrointestinal tract triggers the activation of larvae of *Trichinella* into intestinal infective larvae (IIL). These IIL invade the intestinal epithelium, where they molt and mature into adult worm. The female adult worms release newborn larvae after copulation. Newborn larvae invade host’s capillary vessels and then migrate through the circulatory system to reach the skeletal muscle cells, where they become encapsulated to complete the life cycle [10]. During the life cycle, IIL of *Trichinella* are the initial invasive stage that invades the host’s intestinal epithelial cells (IECs) to establish infection, a process that appears to be regulated by their excretory/secretory products (ESPs) [11]. For example, the putative serine protease and peptidase facilitated the intrusion of IECs and intestinal mucosa by IIL, and immunization of mice with recombinant putative serine protease and peptidase resulted in a 52.70% and 38.6% reduction in adult worm burden, respectively [12,13]. Moreover, our previous research has demonstrated that the invasion of IECs by IIL is promoted by recombinant *T. spiralis* aspartic protease-2 (rTsASP2) and can be inhibited by anti-rTsASP2 serum or siRNA targeting TsASP2. Damage to monolayer IECs caused by IIL invasion was significantly alleviated when larvae treated with siRNA were used [14]. Additionally, mice vaccinated with rTsASP2 showed a 54.17% reduction in adult worms in the intestine and a 54.58% reduction in muscle larvae following challenge with *T. spiralis* [15]. These findings indicate that TsASP2 plays an important role in *T. spiralis* penetration into host IECs. However, the mechanism of the destructive effect of TsASP2 to the gut barrier integrity is not clear.

The intestinal barrier prevents pathogen invasion and restricts antigen and toxin entry into the bloodstream. The gut mucosa, acting as a physical barrier, is formed by IECs and the junctional complex, which includes tight junctions (TJs), adherens junctions, and desmosomes [16,17]. Some protozoa can degrade the intestinal mucosa to enhance their invasion and colonize hosts more efficiently. Recombinant cysteine proteases from *Giardia* and *Entamoeba histolytica* compromise the intestinal epithelial barrier function by degrading TJ proteins (Claudin-1, Claudin-4, Occludin, JAM-1, and β-catenin) and adherens junction protein E-cadherin [18,19]. For intestinal helminths, the dysfunction of the intestinal barrier also provides a more favorable condition for their invasion into IECs, as observed in the case of *T. spiralis*. Studies have showed that ESPs of *T. spiralis* exert a regulatory influence on the junctional proteins of intestinal epithelial cells. Wang et al [20], Li et al [21] and Song et al [22] demonstrated that exosomes and serine proteases in the *T. spiralis* muscle larvae ESPs and IIL ESPs downregulated the expression of Claudin-1, Occludin, and ZO-1, increased the permeability of the epithelial cell monolayer, and improve the worm invasion, but their direct hydrolytic effect was not investigated. In addition, our previous studies indicated that a novel trypsin or serine proteinase from IIL was found to reduce the expression of gut TJ proteins, compromise epithelial integrity and barrier function, and ultimately mediate larval invasion of the gut epithelium by activating the ERK1/2 signaling pathway [16,23]. Moreover, recent study has identified that the *Trichinella spiralis* zinc metalloprotease Nas-14 exhibits direct proteolytic activity against Claudin-1 [24]. However, whether TsASP2 proteases from ESPs directly hydrolyze the junctional proteins of intestinal epithelial cells remains unclear.

Here, in this study, we demonstrated the direct degradation effect of rTsASP2 on TJs *in vitro*. Additionally, we established a protein delivery system using *Lactococcus lactis* and further confirmed the destructive impact of rTsASP2 on the TJs of intestinal epithelial cells *in vivo*. High-Performance Liquid Chromatography Coupled to Time-of-Flight Mass Spectrometry (HPLC-TOF/MS) analysis of the band generated by rTsASP2-mediated hydrolysis of recombinant human Occludin (rhOccludin) specifically identified the cleavage sites as K-S, R-A, R-T, R-E and R-L. This study elucidated the mechanism by which TsASP2 facilitates *T. spiralis* invasion through the hydrolysis of TJ proteins in intestinal epithelial cells, providing critical experimental evidence for understanding host-parasite interactions.

## Materials and methods

### Ethics statement

All of the animal experiments were approved by the Zhengzhou University Life Science Ethics Committee (No. ZZUIRB GZR 2021–0044).

### Experimental animals

Specific pathogen-free, 8-week-old female C57BL/6J mice were purchased from GemPharmatech (Jiangsu, China). They were fed autoclaved food and water.

### Cell, bacterium and parasite

The human colon adenocarcinoma cell line, human colon adenocarcinoma cells (Caco-2), was obtained from the Shanghai Institute for Biological Sciences of the Chinese Academy of Sciences. *Lactococcus lactis* NZ3900 and the plasmid pNZ8149 were provided by Prof. Rongguang Zhang of Zhengzhou University. *Trichinella spiralis* strain ISS534 (T1), the international reference strain of porcine origin isolated from a naturally infected pig in Henan Province, China, was maintained in Kunming mice in our laboratory.

### Preparation of rTsASP2 protein and anti-rTsASP2 antibodies

The rTsASP2 protein was expressed and purified using the pQE-80L/*TsASP2*/BL21 expression system previously established in our laboratory [14], followed by refolding via ultrafiltration and eliminating endotoxins via a high-capacity endotoxin removal resin (Thermo Fisher Scientific, Waltham, USA). The concentration of rTsASP2 protein was quantified using a BCA assay kit (Epizyme, Shanghai). Mice were intraperitoneally immunized with rTsASP2 protein (20 μg) in three doses at 14-day intervals. Two weeks after the final immunization, anti-rTsASP2 serum was obtained through tail-tip blood collection. Approximately 90–100 μL of whole blood can be collected from a C57BL/6J mouse, yielding 35–50 μL of serum following centrifugation.

### Cell culture

Caco-2 cells were maintained in Dulbecco’s Modified Eagle’s Medium (DMEM, Servicebio) supplemented with 10% fetal bovine serum (Sangon Biotech), 100 U/mL penicillin, and 100 μg/mL streptomycin (Sangon Biotech) in T25 flasks (BIOFIL). When cells reached 90% confluence, they were subcultured via trypsin-EDTA digestion [23].

### CCK-8 assay of cell viability

Caco-2 cells were seeded into 96-well cell culture plates and cultured until reaching confluence. 100 μL of DMEM containing 0, 1, 5, 10, 15, 20, 25, or 30 μg/mL rTsASP2 was added respectively. After 2 hours of stimulation, 100 μL of 10% Cell Counting Kit-8 (CCK-8) reagent in DMEM was added to each well. Following a 1-hour incubation, the absorbance at 450 nm (OD₄₅₀) was measured using multi-mode microplate reader (SpectraMax i3X, USA).

### rTsASP2-treated Caco-2 cell lysates

Caco-2 cells were cultured until a confluent monolayer formed. Total cell lysates were extracted using ice-cold Radioimmunoprecipitation Assay (RIPA) buffer (Solarbio) supplemented with Phenylmethylsulfonyl Fluoride (PMSF). Total Caco-2 cell lysates (40 μg) were incubated with 4 μg rTsASP2 at 37 °C for 16 h; denatured rTsASP2 at 100˚C for 15 min was used as a negative control, and PBS as a vehicle control.

### Western blot

First, the protein was separated by sodium dodecyl sulfate polyacrylamide gel electrophoresis (SDS-PAGE) on a 10% acrylamide separation gel and subsequently transferred onto polyvinylidene difluoride (PVDF) membrane. The membrane was blocked with 5% skimmed milk for 2 h at 37˚C and then incubated with antibodies against Occludin (1:1500, Affinity), Claudin-1 (1:1500, Affinity) and glyceraldehyde 3-phosphate dehydrogenase (GAPDH, 1:20000, HUABIO) at 4 ˚C overnight. After washing with TBST, the membrane was incubated with Horseradish Peroxidase (HRP) -conjugated anti-rabbit IgG as secondary antibodies (1:10 000, Abcam). Protein bands were visualized using the Enhanced Pico Light Chemiluminescence Kit (Epizyme, Shanghai) and quantified using Image J.

### rTsASP2-treated Caco-2 monolayer

Caco-2 cells were seeded in 24-well plates at a density of 2 × 10⁵ cells per well and cultured until confluent monolayers formed. Following treatment with 20 μg/mL rTsASP2, PBS (vehicle control), or heat-denatured rTsASP2 (negative control) for 2 h, cellular proteins were lysed and subjected to Western blot analysis to assess TJs changes.

### Assay of transepithelial electrical resistance and paracellular permeability

Caco-2 cells (2 × 10^5^/well) were seeded into 12-well Transwell inserts (LABSELECT). During the culture period, transepithelial electrical resistance (TEER) was monitored using a Millipore ERS-2 epithelial voltohmmeter (Millipore, USA). The following experiments were performed using Caco-2 cell monolayers with a TEER greater than 500 Ω. Prior to cell treatment, TEER was measured. Cells were then treated for 2 hours with one of the following: 20 μg/mL rTsASP2, PBS, or denatured rTsASP2, after which TEER was re-measured to evaluate TEER changes induced by the treatments. To assess paracellular permeability, FITC-dextran of 4 kDa (FD-4) (Sigma, Cat. 46944) was added to the apical chamber simultaneously with the treatments. After incubation for 2 hours, fluid from the basolateral chamber was collected. Fluorescence intensity was measured using multi-mode microplate reader (SpectraMax i3X, USA) at excitation/emission wavelengths of 492/520 nm.

### Construct the recombinant *Lactococcus lactis*- pNZ8149/*TsASP2*/NZ3900

The *TsASP2* gene was amplified by polymerase chain reaction (PCR) from the cDNA of *T. spiralis* muscle larva using the forward primer TAT*CCATGG*CAGGACAAATTCAACCCGTC and the reverse primer CCC*TCTAGA*CGCTAAGAATGTAATTTAGTAGCG; *Nco*I/*Xba*I restriction endonuclease sites were italicized. Then, the PCR product was inserted into the pMD19-T plasmid (Taraka, China) to generate recombinant plasmid pMD19-T/*TsASP2*, which was transformed into *E. coli* DH5α by a heat-shock method [25]. Lastly, the plasmids pNZ8149 and pMD19-T/*TsASP2* were digested with *Nco*I and *Xba*I, ligated with T4 ligase and electro-transformed into competent *L. lactis* NZ3900, which was selected on Elliker culture agar plates. Positive clones were identified by PCR and restriction endonuclease digestion and confirmed by nucleotide sequencing [26].

### Identify the expression of TsASP2 in the recombinant *Lactococcus lactis*

The recombinant *L. lactis* was cultured in M17 broth (hopebio, Qingdao) at 30 °C overnight and then diluted with fresh media at a ratio of 1: 25. When OD_600_ = 0.4, the recombinant *L. lactis* was induced with 25 ng/mL nisin for 5 h. Cells were collected and treated with 10 mg/mL lysozyme for 1 hour and then analyzed by SDS-PAGE and Western blot.

### Characteristics of recombinant *L. lactis*

The biological characteristics of the recombinant *L. lactis* strain, including the growth curve, acid resistance experiment, and genetic stability, were investigated using previously described methods [27].

### Analyze the intestinal colonization capability of recombinant *L. lactis*

C57BL/6J mice were randomly divided into two groups: a normal group and a flora clearance group. The normal group were further subdivided into 3 groups: PBS, pNZ and TsASP2. These subgroups were orally administrated with 200 μL of either PBS, pNZ8149/NZ3900, or pNZ8149/*TsASP2*/NZ3900 (5 × 10^10^ CFU/mL) for 3 consecutive days, respectively. Mice with flora clearance were pretreated with a cocktail of antibiotics (Kanamycin 0.4 mg/mL, Gentamicin 0.035 mg/mL, Colistin 850 U/mL, Metronidazole 0.215 mg/mL, Vancomycin 0.045 mg/mL) in drinking water for 1 week [28,29], they were subsequently freely exposed to sterile water for 3 days and then orally administrated with 200 μL pNZ8149/*TsASP2*/NZ3900 (5 × 10^10^ CFU/mL) for 3 days. Thirty minutes prior to each recombinant bacteria gavage, the mice were administered 5% sodium bicarbonate (pH8.4) via gavage to neutralize stomach acid. The feces and intestinal contents of the mice were collected on the 1st, 3rd, 5th, 7th and 9th days after intragastric administration. Fecal DNA was extracted using Stool Genomic DNA Extraction Kit (Solarbio). PCR was used to detect whether the recombinant *L. lactis* colonized the intestinal tract.

### Immunofluorescence assay

Immunofluorescence staining was performed to evaluate the delivery of TsASP2 to the intestinal mucosa by recombinant stain as described in the previous study [23]. In brief, small intestinal tissues harvested 1- day post- oral gavage were fixed, embedded and sectioned. Following antigen retrieval, tissue sections were blocked with 10% goat serum for 1 h. The sections were then incubated overnight at 4 °C with mouse anti-rTsASP2 serum (1: 100 dilution) as the primary antibody, followed by a 1-h incubation with Fluorescein Isothiocyanate (FITC) -conjugated goat anti-mouse IgG secondary antibody (1:100; Proteintech) at 37 °C. After counterstaining nuclei with 4′,6-diamidino-2-phenylindole (DAPI) (Solarbio), slides were visualized under a fluorescence microscope (SOPTOP).

### *In vivo* assessment of TsASP2 on intestinal barrier integrity

To assess the TsASP2-mediated barrier disruption *in vivo*, 8-week-old female C57BL/6J mice with flora clearance were randomized into three groups with four mice per group: PBS group, pNZ group and TsASP2 group. Bacterial suspensions (200 μL of 5 × 10^10^ CFU/mL in PBS) were administered via oral gavage daily for 5 days. On day 6 post-treatment, mice were euthanized by cervical dislocation. Duodenal tissues were immediately collected for hematoxylin-eosin (H&E) staining and transmission electron microscopy (TEM) analysis.

### Transmission electron microscopy analysis

Tissue specimens (1 mm³) were harvested within 1–3 min post-euthanasia, fixed in EM-grade fixative, and post-fixed in 1% osmium tetroxide with repeated rinsing. After dehydration at 4 °C, Tissue specimens were embedded with pure resin and stepwise polymerization. Ultrathin sections (70 nm) were prepared using a Leica EM UC7 microtome, dual-stained with uranyl acetate and lead citrate, and observed by HT7800 TEM (80KV).

### Measurement of intestinal permeability

On the Day 5, mice were fasted overnight with water deprivation, with four mice per group. The following day, each mouse received an oral gavage of 100 μL FD-4 (50 mg/mL), followed by restored water access. After 4 hours, the mice were euthanized by CO₂ inhalation, blood samples (approximately 300 μL) were collected from the retro-orbital plexus using heparinized glass capillaries with an internal diameter of 0.5 mm. The blood was immediately transferred into EDTA-K2-coated tubes and gently mixed. Plasma was then isolated from the whole blood by centrifugation at 1000 g for 15 min. Fluorescence intensity was measured using multi-mode microplate reader (SpectraMax i3X, USA) at excitation/emission wavelengths of 492/520 nm.

### *In vitro* invasion assay

The Caco-2 cell monolayers were overlaid with IIL suspended in semisolid medium with 20 ug/mL rTsASP2 or denatured rTsASP2 and incubated in 5% CO_2_ for 2 h at 37 °C. PBS group were served as negative control. Invaded larvae within cell monolayers were observed and counted using an inverted phase-contrast microscope (Olympus, Japan). Invaded larvae exhibited active locomotion through the monolayer, while non-invaded larvae remained suspended in the medium in a characteristic spiral coil [22].

### DSS-induced colitis

Four group of mice with flora clearance, pNZ group (n = 6), TsASP2 group (n = 6), pNZ-DSS group (n = 5) and TsASP2-DSS group (n = 5), were used. Mice in the control groups (pNZ and TsASP2 groups) received autoclaved water for the entire 10-day period, while experimental colitis was induced in the DSS groups (pNZ-DSS and TsASP2-DSS groups) by administering a 2.5% (wt/vol) dextran sulfate sodium (DSS, MP Biomedicals, MW 36,000–50,000) solution as drinking water for the first 7 days, followed by autoclaved water for the subsequent 3 days. For the initial 5 days, mice in each group received daily oral administration of 200 μL pNZ8149/NZ3900, or pNZ8149/*TsASP2*/NZ3900 (5 × 10^10^ CFU/mL). On day 10, mice were humanely euthanized via CO₂, with the collection of blood from the retro-orbital plexus, the colon, and the spleen. During the treatment period, all mice were monitored daily for clinical parameters including body weight fluctuations, diarrhea severity, and fecal occult blood. Fecal occult blood was confirmed using Qualitative Fecal Occult Blood Test Kit (YaJi Biological). The disease activity index (DAI) was determined as previously described (S1 Table) [30].

### Histopathological examinations

Colonic tissues were processed using the Swiss-roll technique, fixed, paraffin-embedded, and sectioned for H&E staining. Histopathological evaluation of inflammatory damage severity was performed according to the scoring system established by Wang et al (S2 Table) [30].

### Flow Cytometry

Following euthanasia, retro-orbital blood and spleens were collected. Leukocyte suspensions were prepared for flow cytometric analysis. The dead cells were stained with Zombie Aqua Fixable Viability Kit (Biolegend). Following Fc receptor blocking with anti-mouse CD16/32 antibody (Becton, Dickinson and Company, BD), cells were stained with fluorescently labeled antibodies against CD45 (Biolegend), CD11b (eBioscience), and Ly6G analyzed using a BD FACSCanto Ⅱ flow cytometer (BD Biosciences).

### Molecular Docking

First, we obtained the amino acid sequence of TsASP2 from National Center for Biotechnology Information (NCBI). This sequence was submitted to the AlphaFold Protein Structure Database for retrieval, and the tertiary structure corresponding to the protein model exhibiting 100% sequence identity was selected. For Occludin, its tertiary structure was directly retrieved from the AlphaFold Protein Structure Database. Protein-protein molecular docking was performed using the HDOCK server (http://hdock.phys.hust.edu.cn/) [31, 32,33]. Subsequently, the docked complex was visualized and analyzed using PyMOL Molecular Graphics System.

### Cleavage of rhOccludin by rTsASP2

To further evaluate the hydrolytic activity of rTsASP2, 2 µg of rTsASP2 was incubated with 3 µg of recombinant human Occludin (rhOccludin, Lys266 ~ Thr522, Elabscience, Cat No. PDEH100527) in pH 3 buffer at 37 °C for 16 h. Controls containing rhOccludin alone or rTsASP2 alone were subjected to identical incubation conditions. Subsequently, the hydrolysis products, rhOccludin control, and rTsASP2 control were separated by SDS-PAGE and visualized using a silver staining kit (Servicebio, Wuhan, China).

### High-Performance Liquid Chromatography Coupled to Time-of-Flight Mass Spectrometry

The two major protein bands specific to the hydrolysis products (absent in both the rhOccludin and rTsASP2 controls) were separately excised from the gel and subjected to High-Performance Liquid Chromatography Coupled to Time-of-Flight Mass Spectrometry (HPLC-TOF/MS) analysis by Candidate (Shanghai, China). By comparing the amino acid sequence of each peptide peak with the intact rhOccludin sequence, the specific cleavage sites of rTsASP2 were determined. Theoretical molecular weights of protein fragments were predicted using the ExPASy Compute pI/Mw tool (https://www.expasy.org/resources/compute-pi-mw).

### Statistical analysis

Statistical analyses were performed using GraphPad Prism 8 (GraphPad Software, USA) and Microsoft Excel 2021 (Microsoft Corp., USA). Data are presented as mean ± standard deviation (mean ± SD). Statistical significance was determined using one-way ANOVA, two-way ANOVA, and Student’s t-test where appropriate. A threshold of *P* < 0.05 was considered statistically significant.

## Results

### rTsASP2 directly hydrolyze TJs of intestinal epithelial cells

**Fig 1:**
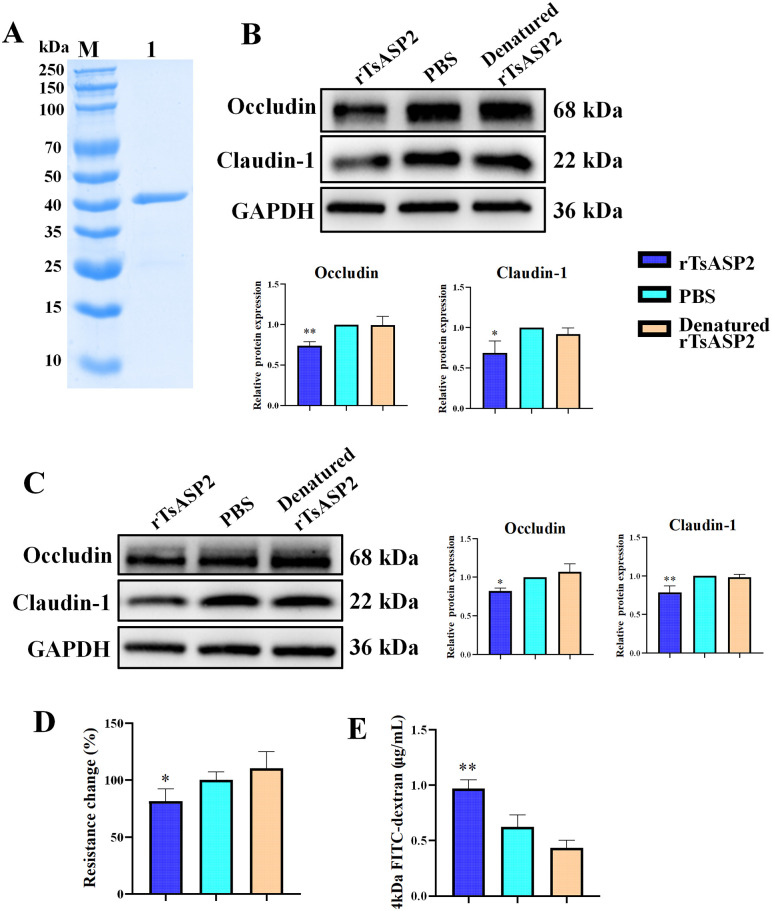
The hydrolysis of TJs in Caco-2 cells by rTsASP2 enhanced paracellular permeability. (**A**) The purified rTsASP2 was analysed by SDS-PAGE. M: protein molecular weight marker; 1: rTsASP2 purified by Ni-NTA Spin Kit. (**B**) The level of Occludin and Claudin-1 in rTsASP2-treated Caco-2 cell lysates was analysed by Western blot and quantified by ImageJ (n = 3). (**C**) The level of Occludin and Claudin-1 in rTsASP2-treated Caco-2 monolayer was analysed by Western blot and quantified by ImageJ. GAPDH served as the loading control (n = 3). (**D**) TEER of cell monolayers pre- and post-rTsASP2 treatment (n = 7–9). (**E**) The permeability of 4 kDa-FITC dextran was increased after Caco-2 monolayer treated with rTsASP2, representative results from one out of three independent experiments with n = 3. The data shown are means ± SD. *P < 0.05, **P < 0.01 versus the PBS group.

The rTsASP2 was expressed in pQE-80L/*TsASP2*/BL21 and purified using Ni-NTA His-tag affinity kit. The SDS-PAGE analysis showed that an individual protein of rTsASP2 with a molecular weight about 43.4 kDa was obtained (**Fig 1A**). After renaturation and eliminating endotoxins, rTsASP2 was processed to incubated with Caco-2 cell lysates at 37 °C for 16 h. Results showed rTsASP2 significantly reduced the levels of Occludin, and Claudin-1 when compared to PBS group or heat-denatured rTsASP2 treated group **(Fig 1B**). Then, we further investigated the effect of rTsASP2 on the hydrolysis of TJs in cultured cells. CCK-8 assay showed that rTsASP2 had no obvious effect on Caco-2 cell viability (**S1 Fig**). Following a 2-hour incubation of the Caco-2 monolayer with rTsASP2, a significant downregulation in the expression of Occludin and Claudin-1 was observed compared to the PBS or heat-denatured rTsASP2 treated group (**Fig 1C**). Besides, after treatment of Caco-2 monolayers with rTsASP2, TEER values were significantly decreased, along with an increase in paracellular permeability to FD-4, as compared to the PBS group or the heat-denatured rTsASP2-treated group (**Fig 1D** and **1E**). These results indicated that rTsASP2 could directly hydrolyze TJs of intestinal epithelial cells and enhanced their paracellular permeability.

### Construction and identification of the recombinant *Lactococcus lactis*

**Fig 2:**
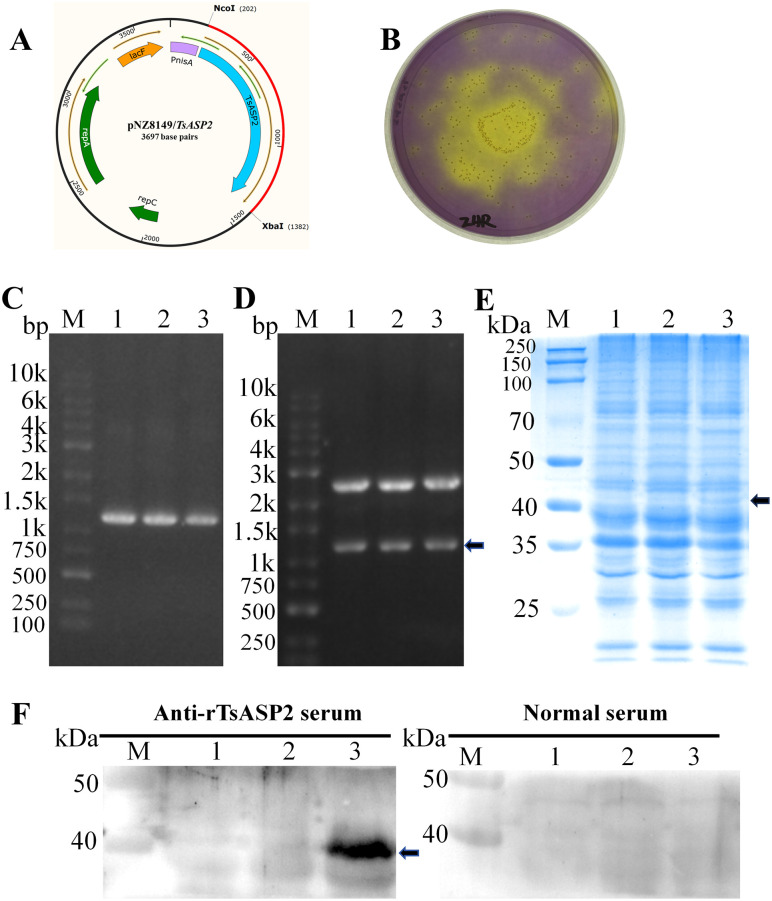
Construction and identification of the recombinant *L. lactis*- pNZ8149/*TsASP2*/NZ3900. (**A**) Schematic of the pNZ8149/*TsASP2* plasmid. (**B**) Positive clones selected as yellow colonies on Elliker selective agar. (**C**) PCR verification of the plasmid pNZ8149/*TsASP2.* M: DNA marker; 1–3: PCR product of *TsASP2*. (**D**) Restriction enzyme digestion verification of the plasmid pNZ8149/*TsASP2.* M: DNA marker; 1–3: digested products of pNZ8149/*TsASP2* with *Nco*I and *Xba*I; the arrow indicated *TsASP2*. (E) SDS-PAGE and (F) Western blot analysis of rTsASP2 expression in recombinant *L. lactis*. SDS-PAGE revealed a protein of approximately 43.4 kDa specifically in recombinant *L. lactis* (E, 3), identified as rTsASP2 by Western blot (F, 3). This band was absent in *L. lactis* with pNZ8149 vector (1) and the non-induced recombinant *L. lactis* (2), confirming successful induction and specific immunodetection. the arrow indicated the rTsASP2.

To directly observe the effect of TsASP2 on IECs *in vivo*, a *L. lactis* expression system was employed to deliver rTsASP2 to the intestine. First, the PCR-amplified *TsASP2* fragment was ligated into the pMD19-T vector and subsequently transformed into *E. coli* DH5α competent cells using heat shock method. Positive clones with recombinant plasmids (pMD19-T/*TsASP2*) were identified through colony PCR amplification of the *TsASP2* (1170 bp) (S2A Fig). The recombinant plasmid was further validated through *Nco*I/*Xba*Ⅰ restriction enzyme digestion, which yielded a large fragment of the same size as pMD19-T and a small fragment of the same size as the *TsASP2* (S2B Fig). The sequencing results confirmed that the insert gene was *TsASP2* fragment sequence (S3 Fig). Then, the target gene generated by double restriction digestion of pMD19-T/*TsASP2* was ligated into the expression vector pNZ8149 (Fig 2A), which was then electroporated into *L. lactis* NZ3900. Positive clones were selected as yellow colonies on Elliker selective agar (Fig 2B). PCR showed that *TsASP2* was successfully ligated into pNZ8149 (Fig 2C), and restriction digestion of the expression recombinant plasmid yielded two fragments corresponding to the expected sizes of *TsASP2* (1,170 bp) and linearized pNZ8149 (2250 bp) (Fig 2D). Finally, the expression of rTsASP2 in the positive recombinant *L. lactis* (pNZ8149/*TsASP2*/NZ3900) was separated by SDS-PAGE and identified by Western blot. Anti-rTsASP2 serum, obtained by immunizing mice with purified rTsASP2 (S4 Fig), was used for detection. Results showed that there was an appropriate-sized protein (about 43.4 kDa) was expressed (Fig 2E) when the recombinant *L. lactis* induced by nisin, that could be identified by anti-rTsASP2 serum (Fig 2F). These results confirmed the successful construction of the recombinant *L. lactis*- pNZ8149/*TsASP2*/NZ3900.

### Biological characteristics of recombinant *L. lactis*

**Fig 3:**
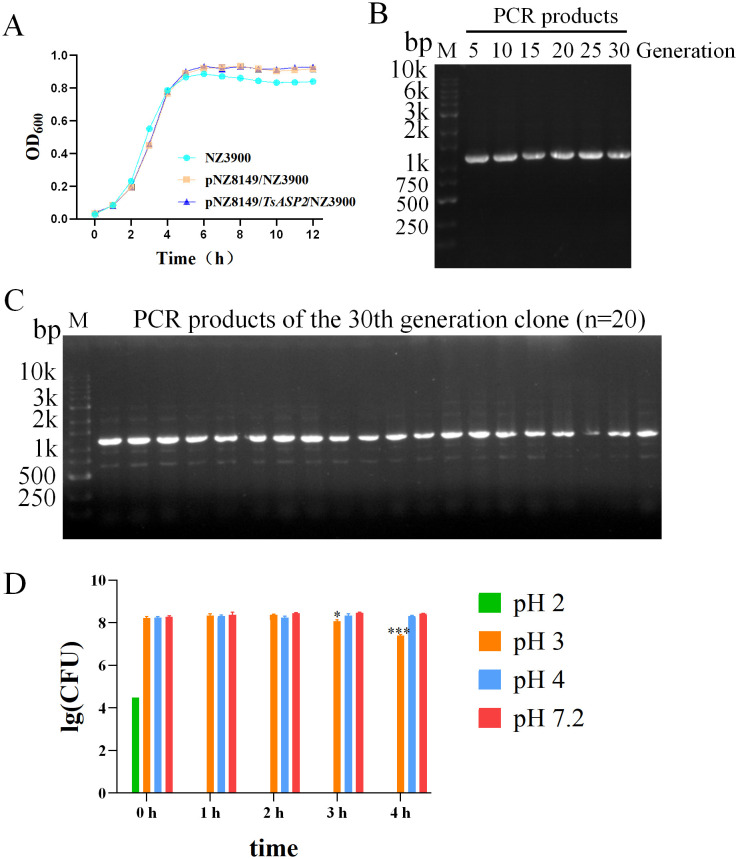
Biological characteristics of recombinant *L. lactis.* (**A**) The growth curves of NZ3900, pNZ8149/NZ3900, and pNZ8149/*TsASP2*/NZ3900. Data are shown as mean ± SD (n = 3). (**B**) PCR products of 5, 10, 15, 20, 25, 30 generations of recombinant *L. lactis.* M: DNA marker. (**C**) PCR products from 20 individual colonies of the 30th generation. M: DNA marker. (**D**) Tolerance of recombinant *L. lactis* in acid environment. Data are shown as mean ± SD (n = 3). Results were from one of two experiments performed showing similar results.

Growth curves were generated to analyze whether *TsASP2* implantation affects cellular growth. Results demonstrated that NZ3900, pNZ8149/NZ3900, and pNZ8149/*TsASP2*/NZ3900 exhibited slow growth during the first 2 hours. Then they entered the logarithmic growth phase over the following 5 hours. After approximately 7 hours, bacterial growth transitioned into the stationary phase. Notably, the growth pattern of pNZ8149/*TsASP2*/NZ3900 aligned with those of pNZ8149/NZ3900 and NZ3900 (**Fig 3A**), indicating that the introduction of recombinant plasmids did not impact the host strain’s growth. To further investigate whether the recombinant plasmids can be stably maintained and inherited in cells, a strain subculture experiment was conducted. Results demonstrated that *TsASP2* was consistently detected in recombinant *L. lactis* over 30 consecutive generations, with a 100% plasmid positivity rate observed in the 30th-generation colonies, indicating stable genetic inheritance (**Fig 3B** and **3C**). Moreover, considering the strongly acidic nature of gastric fluid, an *in vitro* acid tolerance test was conducted prior to intragastric administration to evaluate the survival capability of recombinant *L. lactis* under varying pH conditions. Results revealed that recombinant *L. lactis* exhibited no viability in highly acidic environments (pH 1.0-2.0), while maintaining survival capability for 4 hours in digestive fluids at pH 3, pH 4, and pH 7.2. However, a marked reduction in colony counts was observed after 3 and 4 hours of incubation in pH 3 digestive fluid (**Fig 3D**). Therefore, to ensure the viability of the cells, in the subsequent experiments, 5% sodium bicarbonate was used to administer mice half an hour before gavage with the recombinant *L. lactis*.

### Colonization characteristics of recombinant *L. lactis*

**Fig 4:**
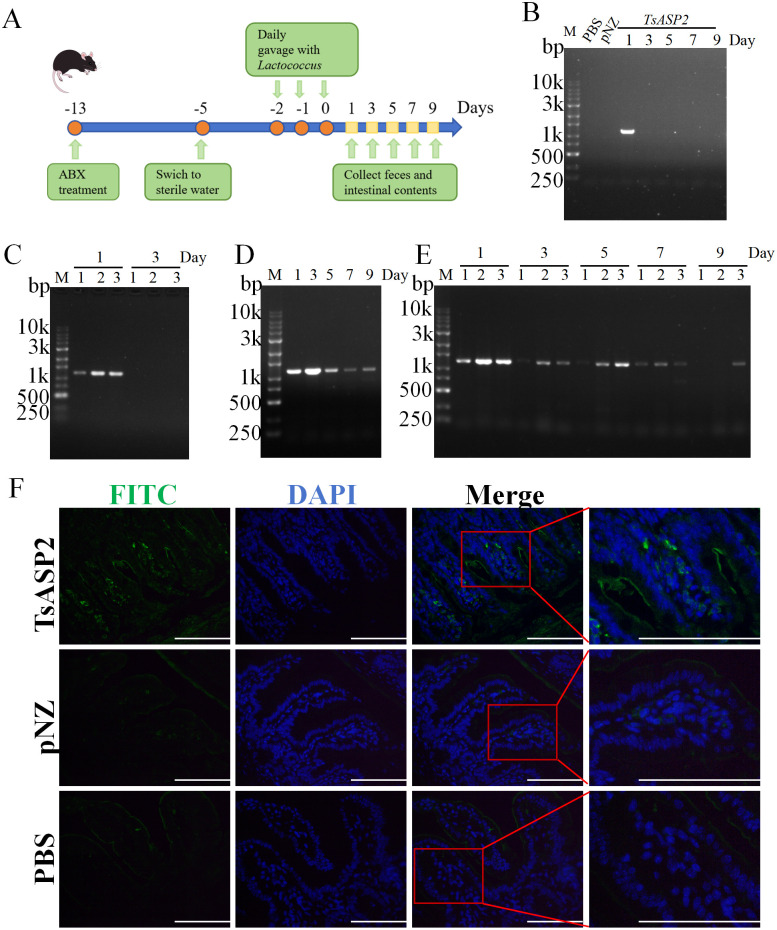
Colonization characteristics of recombinant *L. lactis.* (**A**) Schematic of recombinant *L. lactis-* treated and sample- collected process. (**B**) PCR products amplified from fecal DNA of normal mice. (**C**) PCR products amplified from ileal (1), cecal (2), and colonic (3) contents DNA of normal mice. (**D**) PCR products amplified from fecal DNA of mice with flora clearance. (**E**) PCR products amplified from ileal (1), cecal (2), and colonic (3) contents DNA of mice with flora clearance. (**F**) Positive green fluorescence staining was clearly observed in intestinal epithelium of mice immunized with recombinant *L. lactis*. There was no positive fluorescence staining in two control groups. The nuclei of intestinal cells were stained blue by DAPI. Scale bar = 100 μm. Results were from one of three experiments performed showing similar results.

To determine whether the recombinant bacteria could colonize *in vivo*, we administered them via gavage for three consecutive days in both normal mice and mice with flora clearance, followed by PCR assessment of *TsASP2* in intestinal contents and fecal samples at various time points (**Fig 4A**). Results revealed that recombinant *L. lactis* failed to colonize in normal mice, as the target gene- *TsASP2* was transiently detected in intestinal luminal samples and feces exclusively on day 1, with complete clearance by day 3 (**Fig 4B** and **4C**). While in mice with flora clearance, the recombinant *L. lactis* maintained stable colonization for 7 days, with *TsASP2* detected in fecal samples, cecal contents, and colonic contents throughout this period (**Fig 4D** and **4E**). The target gene became undetectable in cecal contents of mice with flora clearance by day 9, marking the onset of bacterial clearance (**Fig 4D** and **4E**). Subsequently, we further analyzed the ability of the recombinant *L. lactis* to express rTsASP2 *in vivo*. Intestinal tissues were collected one day after gavage with the recombinant *L. lactis*, and the rTsASP2 in the intestinal mucosa was detected by IFA. Results indicated that rTsASP2 was observed in intestinal epithelium of mice treated with recombinant *L. lactis* (**Fig 4F**). Furthermore, expression of rTsASP2 in the intestinal epithelium was still detectable on the fifth day post-gavage (**S5 Fig**). No fluorescence staining was detected in intestinal sections from PBS-treated or pNZ8149/NZ3900- treated mice (**Fig 4F**).

### Recombinant *L. lactis* destroy the intestinal barrier

**Fig 5:**
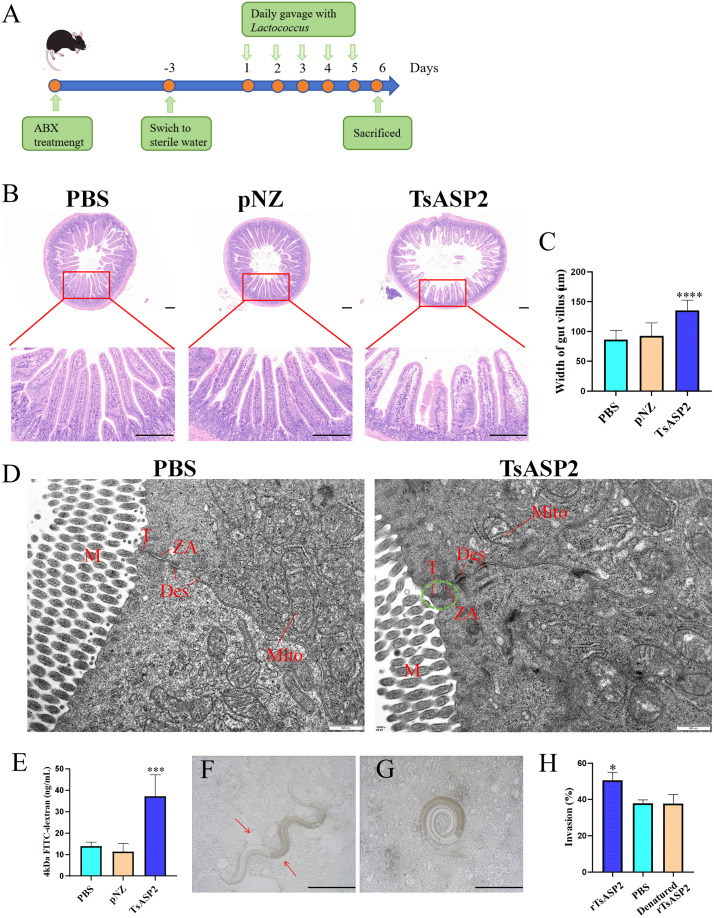
Recombinant *L. lactis* destroy the intestinal barrier. (**A**) Schematic of recombinant *L. lactis-* treated process. (**B**) The histopathological changes in the duodenum were examined by H&E staining. Scale bars = 200 μm. (**C**) Width of gut villus in each high-power field was determined using the ImageJ software for the duodenum samples. Data shown are means ± SD (n = 4) from one of two experiments performed showing similar results. (**D**) Transmission electron microscopy was used to observe the junction complex between intestinal epithelial cells. T: tight junction, ZA: intermediate junction, Des: desmosome, M: microvilli, Mito: mitochondria. Damaged T and ZA were indicated by green dashed circles. Scale bars = 500 nm. (**E**) Mice were gavaged with 50 mg/mL FD4, and its level in the serum was assessed 4 hours later. Data shown are means ± SD (n = 3–4) from one of two experiments performed showing similar results. (**F**) Invaded larvae in the monolayer. Arrows indicated the larval migratory traces. (**G**) Non-invaded larvae. Scale bars = 100 μm. (**H**) Facilitation of rTsASP2 on larval invasion. Data shown are means ± SD (n = 3) from one of two experiments performed showing similar results. *P < 0.05, ***P < 0.001, ****P < 0.0001 versus the PBS group. A-E: PBS indicates PBS treated; pNZ indicates pNZ8149/NZ3900 treated; TsASP2 indicates pNZ8149/*TsASP2*/NZ3900 treated.

From the aforementioned data, it has been demonstrated that recombinant *L. lactis* can successfully colonize the intestinal mucosa and express rTsASP2. To investigate the effects of TsASP2 on the intestinal mucosa, we harvested intestinal tissues from mice treated with recombinant *L. lactis* and performed pathological analyses (**Fig 5A**). H&E staining revealed architectural disruption of intestinal villi in tissue sections from the mice in TsASP2 group compared to tissue sections from the mice in PBS group, characterized by villous edema and thickening (**Fig 5B** and **5C**). Compared with the PBS group, there were no obvious changes in the intestinal tissues of mice from the pNZ group. In addition, Transmission electron microscopy was further used to assess the disruption of TJs between mouse epithelial cells caused by rTsASP2. Results revealed that intestinal tissues from the PBS group maintained intact TJs, adherens junctions, and desmosomes. In contrast, tissues from the TsASP2 group exhibited structural disorganization of tight and adherens junctions, accompanied by mitochondrial ultrastructural damage (**Fig 5D**). What’s more, the intestinal permeability assay revealed that intestinal permeability of the TsASP2 groups was increased compared to PBS group (**Fig 5E**). Subsequently, *in vitro* invasion assays demonstrated that rTsASP2 enhanced IIL invasion into Caco-2 cell monolayers compared to the PBS control group, whereas inactivated rTsASP2 failed to facilitate invasion (**Fig 5F-H)**. These findings demonstrated that TsASP2 disrupted intercellular junctional complexes in intestinal epithelial cells, increased intestinal mucosal permeability, compromised the integrity and barrier function of the intestinal mucosa and enhanced larval invasion. PBS indicates PBS treated; pNZ indicates pNZ8149/NZ3900 treated; TsASP2 indicates pNZ8149/*TsASP2*/NZ3900 treated.

### Recombinant *L. lactis* exacerbated DSS-induced colitis

**Fig 6:**
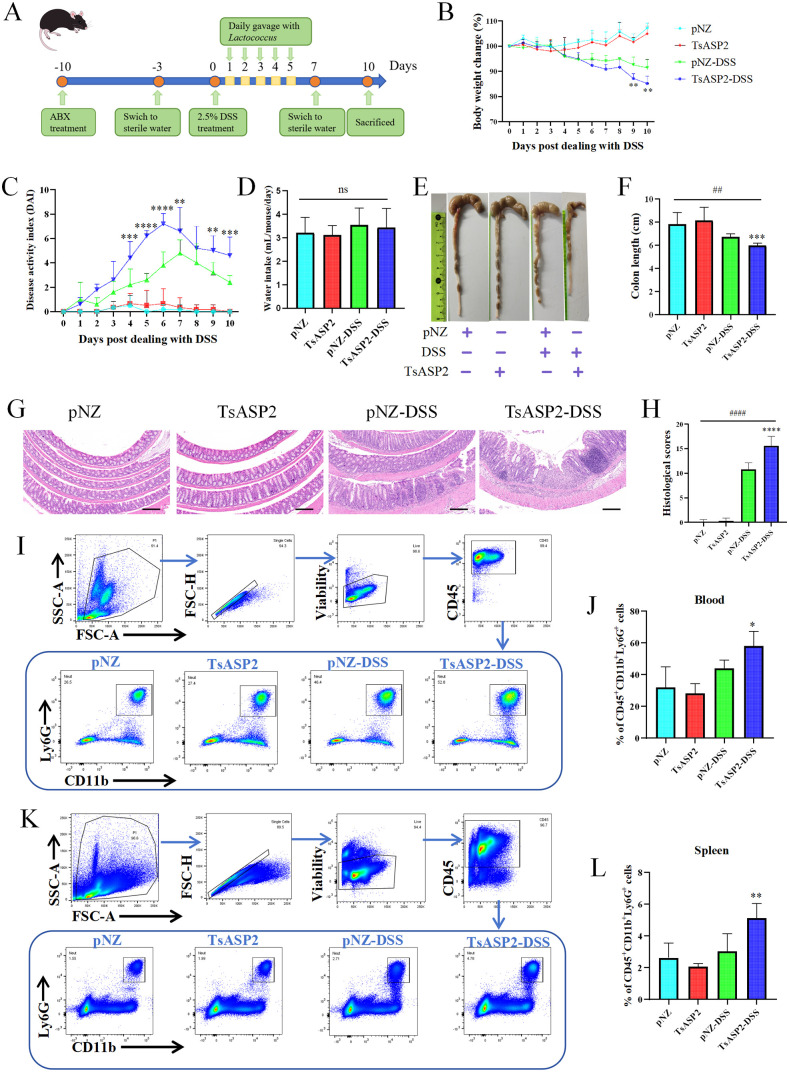
Recombinant *L. lactis* exacerbated DSS-induced colitis. (**A**) Schedule of recombinant *L. lactis-* treated and DSS-induced colitis process. (**B**) Weight change in percent. (**C**) The changes in disease activity index (DAI), which included diarrhea, bleeding and body weight loss. (**D**) Average daily water intake per group of mice during DSS treatment. (**E**) Macroscopic appearance of the colon. (**F**) The colon length in each group. (**G**) The histopathological changes in the colon tissues were examined by H&E staining. Scale bars = 200 μm. (**H**) Histopathological scores were determined for the colon tissue samples. (**I**) Gating strategy of peripheral blood (CD45^+^Ly6G^+^CD11b^+^) in flow cytometry analysis (FACS). (**J**) The percentage of neutrophils in peripheral blood. (**K**) Gating strategy of spleen in FACS. (**L**)The percentage of neutrophils in spleen. The data shown are means ± SD. Representative results from one out of two independent experiments with n = 5–6. *P < 0.05, **P < 0.01, ***P < 0.001, ****P < 0.0001 versus the pNZ-DSS group; #*P* < 0.05, ##*P* < 0.01, ####*P* < 0.0001 versus the respective control group; ns: no significant difference. pNZ indicates pNZ8149/NZ3900 treated; TsASP2 indicates pNZ8149/*TsASP2*/NZ3900 treated; pNZ-DSS indicates pNZ8149/NZ3900 treated and DSS induced colitis; TsASP2-DSS indicates pNZ8149/*TsASP2*/NZ3900 treated and DSS induced colitis.

The damage to the intestinal barrier may aggravate inflammatory bowel disease [34]. Then, a colitis mouse mode was established by administering 2.5% DSS through drinking water to future evaluate the damage caused by rTsASP2 to the intestinal barrier (**Fig 6A**). As shown in Fig 6B and 6C, DSS-treated groups exhibited marked inflammatory signs, characterized by weight loss, rectal bleeding, and diarrhea, which led to a significant elevation in DAI. Mice receiving oral gavage of recombinant *L. lactis* showed exacerbated weight loss and disease symptoms in DSS- induced colitis when compared to mice receiving oral gavage of pNZ8148/NZ3900 (**Fig 6B** and **6C)**. Daily water intake measurement showed that mice were exposed to a similar volume of DSS water in both the TsASP2-DSS group and the pNZ-DSS group (**Fig 6D**). Meanwhile, colonic shortening was more pronounced in the TsASP2-DSS group compared with the pNZ-DSS group (**Fig 6E** and **6F)**. Microscopic examination revealed that DSS- treated mice exhibited typical pathological changes, including epithelial erosion, edema, loss of the mucus layer, substantial polymorphonuclear cell infiltration into the lamina propria, and increased histopathological scores, and mice treated with recombinant *L. lactis* exhibited exacerbated pathological changes compared to those receiving oral administration of pNZ8148/NZ3900 (**Fig 6G** and **6H**). *L. lactis* with pNZ8148 or *TsASP2* recombinant pNZ8148 treatment alone mice showed no weight loss or microscopic damage of the colon. In addition, as the degree of neutrophil infiltration is closely correlated with disease activity in inflammatory bowel disease [35], neutrophils in the peripheral blood and spleens of mice were assessed by flow cytometry. Results indicated that compared with the pNZ-DSS group, mice in the DSS group receiving oral gavage of recombinant *L. lactis* exhibited a significant increase in the percentage of neutrophil in both the spleen and peripheral blood (**Fig 6I- L**). These results demonstrated that recombinant *L. lactis* expressing TsASP2 exacerbated DSS-induced colitis.

### Proteolytic activity of rTsASP2 on rhOccludin

**Fig 7:**
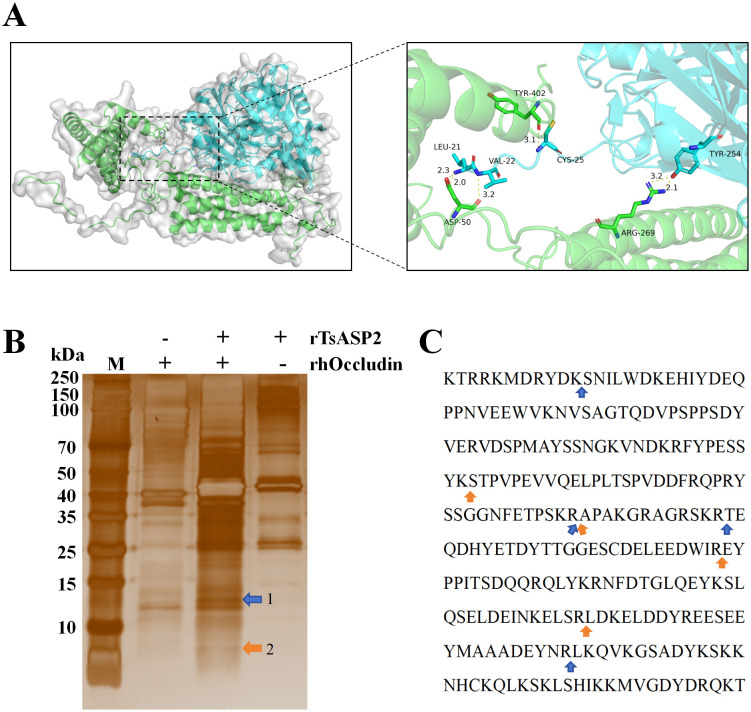
Proteolytic activity of rTsASP2 on rhOccludin. (**A**) Molecular docking model of TsASP2-Occludin interaction. TsASP2 was shown in cyan and Occludin in green; key interfacial residues were represented as sticks within the dashed box; hydrogen bonds were indicated by yellow dashed lines. (**B**) SDS-PAGE analysis of the co-incubation protein products of rTsASP and rhOccludin. M: protein molecular weight marker. Arrows indicated the two major protein bands specific to the hydrolysis products (absent in both the rhOccludin and rTsASP2 controls): the blue arrow indicated Band 1, and the orange arrow indicated Band 2. (**C**) Mapping rTsASP2 cleavage sites on rhOccludin. Black text denoted rhOccludin amino acid sequence; the blue arrow indicated the hydrolysis site identified by integrating sequencing data from Band 1; the orange arrow indicated the hydrolysis site identified by integrating sequencing data from Band 2.

To analyse the effect of TsASP2 on junctional proteins, protein-protein molecular docking was performed using the HDock server. The predicted result indicated a strong binding interaction between TsASP2 and Occludin, as evidenced by a docking score of -290.27. The associated confidence score of 0.9430 further validated the stability of the docked pose (Fig 7A and S3 Table). To further understand the hydrolysis of rTsASP2 on rhOccludin, their co-incubation products were analyzed by SDS-PAGE and subjected to HPLC-TOF/MS. SDS-PAGE of the co-incubation protein products of rTsASP2 and rhOccludin revealed that the hydrolysis products exhibited two additional distinct protein bands, compared with the rhOccludin control and rTsASP2 control groups (Fig 7B). These two unique bands were subjected to HPLC-TOF/MS analysis, which confirmed the presence of partial peptide fragments derived from rhOccludin in all bands, indicating that rTsASP2 proteolytically cleaves rhOccludin. Subsequently, the rhOccludin-derived fragments from the protein bands were reassembled based on mass spectrometry data, and it was speculated that rTsASP2 hydrolyzed peptide bonds between K-S, R-A, R-T, R-E and R-L residues in rhOccludin (Fig 7C and Table 1). The molecular weights of the amino acid sequences spanning the cleavage sites between K-S and R-A, between R-T and R-L, and between R-E and R-L, predicted using the ExPASy Compute pI/Mw tool, were slightly lower than the apparent molecular weight of Band (as determined by SDS-PAGE) (Fig 7B and Table 1).

**Table 1:** Sequences of peptides of rhOccludin generated by hydrolysis using rTsASP2.

Band	Peptide Sequence (MS-detected)	Amino Acid Sequence (Reconstructed)	Predicted MW
1	NVSAGTQDVPSPPSDYVER	SNILWDKEHIYDEQPPNVEEWVKNVSAGTQDVPSPPSDYVERVDSPMAYSSNGKVNDKRFYPESSYKSTPVPEVVQELPLTSPVDDFRQPRYSSGGNFETPSKR	11.822 kDa
	VDSPMAYSSNGK		
	YSSGGNFETPSKR		
	STPVPEVVQELPLTSPVDDFR		
	SNILWDKEHIYDEQPPNVEEWVK		
	EHIYDEQPPNVEEWVK		
	FYPESSYK		
	NFDTGLQEYK	TEQDHYETDYTTGGESCDELEEDWIREYPPITSDQQRQLYKRNFDTGLQEYKSLQSELDEINKELSRLDKELDDYREESEEYMAAADEYNR	11.003 kDa
	SLQSELDEINKELSR		
	SLQSELDEINK		
	TEQDHYETDYTTGGESCDELEEDWIR		
	ELDDYREESEEYMAAADEYNR		
	LDKELDDYREESEEYMAAADEYNR		
	RNFDTGLQEYK		
	LDKELDDYR		
	EYPPITSDQQR		
2	STPVPEVVQELPLTSPVDDFRQPR	STPVPEVVQELPLTSPVDDFRQPRYSSGGNFETPSKR	4.118 kDa
	STPVPEVVQELPLTSPVDDFR		
	YSSGGNFETPSKR		
	YSSGGNFETPSK		
	SLQSELDEINKELSR	EYPPITSDQQRQLYKRNFDTGLQEYKSLQSELDEINKELSR	4.961 kDa
	SLQSELDEINK		
	NFDTGLQEYK		
	EYPPITSDQQR		

## Discussion

Trichinosis is a worldwide zoonotic disease by a nematode *Trichinella* spp. Human infection is acquired through ingestion of undercooked meat containing infective larvae [36]. Following ingestion of infected meat, *T. spiralis* muscle larvae (ML) are released from their capsules in the stomach and are activated by intestinal contents or bile into IIL. After invading the intestinal epithelial cells, the IIL develops into the adult stage through four molts. Thus, the invasion of the intestinal epithelium by the IIL and its subsequent development represent a critical step in *T. spiralis* infection of the host [37,38]. The integrity of the intestinal epithelial barrier is a vital defense against pathogen invasion. ESPs from the IIL stage of *T. spiralis* disrupt barrier function through multiple signal pathways: serine protease (rTsSPc) binding to RACK1 activates the MAPK/ERK1/2 pathway, trypsin (rTsTryp) activating the PAR2 receptor also triggers the ERK1/2 pathway, and C-type lectin (rTsCTL) binding to Syndecan-1 activates the STAT3 pathway. Activation of these pathways downregulates key junctional proteins like Occludin, Claudin-1, and E-cadherin, upregulates Claudin-2, weakens the barrier, and aids larval invasion [16,22,23,39].

Aspartic proteases are widely distributed in parasites. As proteolytic enzymes, their hydrolytic activity toward host proteins plays critical roles in parasite nutrient acquisition, host invasion, development and growth, immune regulation, and aging [40–42]. For instance, the aspartic proteases of *Demodex folliculorum* (Df.ASP) and *Demodex brevis* (Db.ASP) hydrolyze 38 and 23 distinct protein substrates, respectively, in human immortalized keratinocytes (HaCaT cells), causing dermal damage [43]. Similarly, aspartic proteases in hookworms, *Schistosomes*, and *Plasmodium* species degrade hemoglobin to provide nutrients for the parasites [44,45]. Previous studies in our laboratory have shown that TsASP2 exhibits its highest transcriptional expression level during the IIL stage. Present in ESPs, this enzyme demonstrates hydrolytic activity against hemoglobin, collagen IV, and human IgM, and facilitates the invasion of intestinal epithelial cells by the parasite [14]. However, the specific invasion mechanism remains to be elucidated. In this study, we reveal that TsASP disrupts tight junctions by hydrolyzing the peptide bonds between -S, R-A, R-T, R-E, and R-L residues of tight junction proteins, compromising the intestinal barrier, and facilitating the invasion of *Trichinella spiralis* larvae into intestinal epithelial cells. The gastrointestinal epithelial barrier consists of the epithelial cell layer, intercellular junctional complexes, the mucus barrier, and gut microbiota [46]. TJs include Occludin, Claudins, ZO, JAMs, and Zonulin. They are connected to the cytoskeleton and determine the permeability and selectivity of the epithelial layer [47]. Claudins are a large family of tetraspanning integral membrane proteins that are uniquely responsible for the selective permeability of TJs [48]. As the first identified tight junction transmembrane protein, Occludin disruption increases intestinal permeability to macromolecules [49]. In this study, direct hydrolysis assays in Caco-2 cell lysates demonstrated that rTsASP2 cleaved Occludin and Claudin-1. Consistent with this finding, Western blot analysis of rTsASP2- treated Caco-2 monolayers revealed comparable degradation of both TJs. A recent study also reported that while the *Trichinella spiralis* zinc metalloprotease Nas-14 downregulated Claudin-1 and Occludin in treated Caco-2 cells, it specifically degraded only Claudin-1, and not Occludin, when incubated with Caco-2 cell lysates [24]. The complete polarized Caco-2 cells resemble human small intestinal mucosa cells expressing brush borders, TJs and, efflux and uptake transporters at both apical and basolateral compartments [50]. Using Transwell inserts, Caco-2 cells were cultured to model the small intestinal epithelial barrier. Following treatment with rTsASP2, a significant reduction in TEER and increased permeability to FD-4 were observed, indicating disruption of paracellular barrier function. Collectively, these findings demonstrate that rTsASP2 directly targets and degrades key TJs. Notably, this TJ-targeting strategy converges with mechanisms employed by other enteric parasites. For example, *Giardia lamblia* cysteine proteases hydrolyze E-cadherin and Occludin to disrupt epithelial barriers [18]; *Entamoeba histolytica* cystein protease (rEhCP112) has affinity to claudin-1 and claudin-2 and it degrades and delocalizes both TJs [19].

Here, the *L. lactis* NZ3900 expression system was used to deliver rTsASP2 to the intestine for observing its effects on TJs *in vivo.* Our results demonstrate the potential and limitations of using recombinant *L. lactis* for targeted intestinal antigen delivery. While recombinant *L. lactis* exhibited favorable *in vitro* characteristics such as plasmid stability and tolerance to mild gastric acidity (pH 3–4), its *in vivo* persistence was critically dependent on the gut microbiota status. Consistent with previous reports on the colonization studies of *L. lactis* strains [51], we observed that the recombinant *L. lactis* and its target gene were rapidly cleared within 72 hours from the gastrointestinal tracts of mice with intact microbiota. This clearance is attributable to colonization resistance against foreign strains by the commensal community [52]. Crucially, this barrier was overcome in microbiota-depleted mice. Following antibiotic pre-treatment [28], the recombinant *L. lactis* achieved sustained colonization for at least 7 days, primarily evidenced by persistent detection of the target gene in intestinal contents and feces. Immunofluorescence analysis detected recombinant protein in the small intestine. Furthermore, it has been reported that following oral gavage with *L. lactis* NZ3900 expressing human FGF21, the protein was successfully detected in mouse serum by ELISA [53]. This successful colonization enabled the effective delivery of TsASP2 to the intestinal mucosa, thereby allowing us to model the effects of this antigen during simulated parasite invasion. Subsequent experiments with the recombinant *L. lactis* demonstrated that rTsASP2 directly compromises intestinal epithelial integrity through the targeted disruption of apical junctional complexes. The observed villous damage (swelling) and ultrastructural dissolution of TJs provide mechanistic evidence for TsASP2 as a virulence effector of *T. spiralis*. By inducing paracellular hyperpermeability, rTsASP2 likely facilitates parasite invasion of the intestinal mucosa. In other studies, intraperitoneal injection of recombinant *T. spiralis* C-type lectin has been observed to cause villous swelling, inflammatory cell infiltration, downregulation of TJs expression, and increased intestinal permeability [39]. These *in vivo* findings align with our *in vitro* results, consistently demonstrating that rTsASP2 compromises intestinal barrier integrity through disruption of epithelial TJs. This junctional degradation increases paracellular permeability, thereby facilitating larval invasion.

Moreover, accumulating evidence indicates that increased intestinal permeability plays a pathogenic role in inflammatory bowel disease (IBD), celiac disease, and functional bowel disorders such as irritable bowel syndrome [54]. IBD is a chronic inflammatory disorder of the gastrointestinal tract, clinically encompassing Crohn’s disease (CD) and ulcerative colitis (UC). Disruption of intestinal epithelial TJs by any pathogen can promote IBD progression through enhanced gut permeability [34]. Building on this pathogenic framework, we employed a chemically induced colitis model to investigate the intestinal barrier-disrupting effects of TsASP2. We provided compelling evidence that rTsASP2 significantly aggravates disease severity. Recombinant *L. lactis*- treated mice exhibited accelerated weight loss, increased fecal occult blood, greater colon shortening, and elevated disease activity indices, alongside exacerbated histopathological damage (e.g., crypt destruction, goblet cell depletion, mucosal edema) and systemic neutrophilia. This profound exacerbation strongly suggests that the TJs degradation and consequent barrier hyperpermeability induced by TsASP2 drive inflammatory pathogenesis.

Additionally, this study investigated the hydrolysis sites of rTsASP2 on TJ proteins. Occludin, which has a higher molecular weight (68 kDa) compared to Claudin-1 (22 kDa), was selected for this analysis. HDock analysis revealed a strong binding interaction between TsASP2 and Occludin, exhibiting strong binding (docking score: -290.27) and complex stability (confidence score: 0.9430). This finding was strongly corroborated by subsequent enzymatic digestion assays. SDS-PAGE analysis of the products from co-incubation of rTsASP2 with rhOccludin displayed two distinct bands absent in both the rhOccludin-only and rTsASP2-only control groups. HPLC-TOF/MS identification confirmed these bands as rhOccludin-derived peptides, demonstrating that rTsASP2 possesses proteolytic cleavage activity against rhOccludin. Through HPLC-TOF/MS analysis of bands derived from rTsASP2-digested rhOccludin, the cleavage sites were identified as K-S, R-A, R-T, R-E and R-L. It has been reported that aspartic proteases have broad specificity and exhibit a cleavage preference for hydrophobic/aromatic amino acid residue [55,56]. Also, HPLC-TOF/MS has certain limitations; it can only analyze peptides after enzymatic digestion, not the full-length proteins of Band 1 and Band 2. The observed discrepancy, where the molecular weight calculated from the predicted amino acid sequence is lower than the experimentally determined value, may be attributed to post-translational modifications (PTMs) [57].

In conclusion, our findings demonstrate that TsASP2 acts as a critical virulence protease that destabilizes intestinal barrier integrity through hydrolysis of epithelial tight junction proteins sites between K-S, R-A, R-T, R-E and R-L residues (e.g., Occludin, Claudin-1), thereby increasing mucosal permeability and compromising barrier function, which facilitates larval invasion of *T. spiralis*.

## Supporting information

S1 Table Assessment of disease activity index (DAI). (DOCX)

S2 Table Assessment of histopathological scores. (DOCX)

S3 Table Docking assessment of Occludin and TsASP2. (DOCX)

S1 Fig CCK-8 assay for the viability of Caco-2 cells treated with rTsASP2. (DOCX)

S2 Fig Verification of the recombinant pMD19-T/*TsASP2*/DH5 α.
(DOCX)

S3 Fig Comparison of recombination plasmid sequencing. (DOCX)

S4 Fig Serum antibody titer in rTsASP2-immunized mice. (DOCX)

S5 Fig The recombinant *L. lactis* expressing rTsASP2 in the colonic epithelium. (DOCX)
