# Supplementary material for: Aspartic protease 2 from Trichinella spiralis excretion/secretion products hydrolyzes tight junctions of intestinal epithelial cells
Source: PLoS Negl Trop Dis. 2025 Dec 8;19(12):e0013805. doi: 10.1371/journal.pntd.0013805 (PMC12700411; doi:10.1371/journal.pntd.0013805)
Supplement: S1 Table — (DOCX) [file pntd.0013805.s001.docx]

**S1 Table: Assessment of disease activity index (DAI)**

| Score | Body weight loss (%) | Stool | Bleeding |
| --- | --- | --- | --- |
| 0 | <2 | Normal | No color change (2 min) |
| 1 | ≥2 - <5 | Softer stool | Colorless→light purple |
| 2 | ≥5 - <10 | Moderate diarrhoea | Light purple→distinct purple |
| 3 | ≥10 - <15 | diarrhoea | Immediate purple→dark purple |
| 4 | ≥15 | - | Immediate dark purple/bloody stool/anal bleeding |
