# Supplementary material for: Aspartic protease 2 from Trichinella spiralis excretion/secretion products hydrolyzes tight junctions of intestinal epithelial cells
Source: PLoS Negl Trop Dis. 2025 Dec 8;19(12):e0013805. doi: 10.1371/journal.pntd.0013805 (PMC12700411; doi:10.1371/journal.pntd.0013805)
Supplement: S2 Table — (DOCX) [file pntd.0013805.s002.docx]

**S2 Table: Assessment of histopathological scores**

| Score | Extent of inflammation Infiltration | Neutrophils + lymphohistiocytes | Extent of crypt damage | Mucosal oedema | Loss of goblet cells |
| --- | --- | --- | --- | --- | --- |
| 0 | None | None | None | None | None |
| 1 | Mucosa | Focal | One third | Focal | Focal |
| 2 | Mucosa+submucosa | Multifocal | Two thirds | Multifocal | Multifocal |
| 3 | Mucosa+submucosa+muscle layer | Diffuse | Entire crypt damage | Diffuse | Diffuse |
| 4 | Transmura |  | Crypt damage+ulceration |  |  |
