# Supplementary material for: Aspartic protease 2 from Trichinella spiralis excretion/secretion products hydrolyzes tight junctions of intestinal epithelial cells
Source: PLoS Negl Trop Dis. 2025 Dec 8;19(12):e0013805. doi: 10.1371/journal.pntd.0013805 (PMC12700411; doi:10.1371/journal.pntd.0013805)
Supplement: S3 Table — (DOCX) [file pntd.0013805.s003.docx]

**S3 Table Docking assessment of Occludin and TsASP2**

| Docking Assessment | |
| --- | --- |
| Docking Score | -290.27 |
| Confidence Score | 0.9430 |
