# Supplementary material for: Aspartic protease 2 from Trichinella spiralis excretion/secretion products hydrolyzes tight junctions of intestinal epithelial cells
Source: PLoS Negl Trop Dis. 2025 Dec 8;19(12):e0013805. doi: 10.1371/journal.pntd.0013805 (PMC12700411; doi:10.1371/journal.pntd.0013805)
Supplement: S1 Fig — (DOCX) [file pntd.0013805.s004.docx]

**
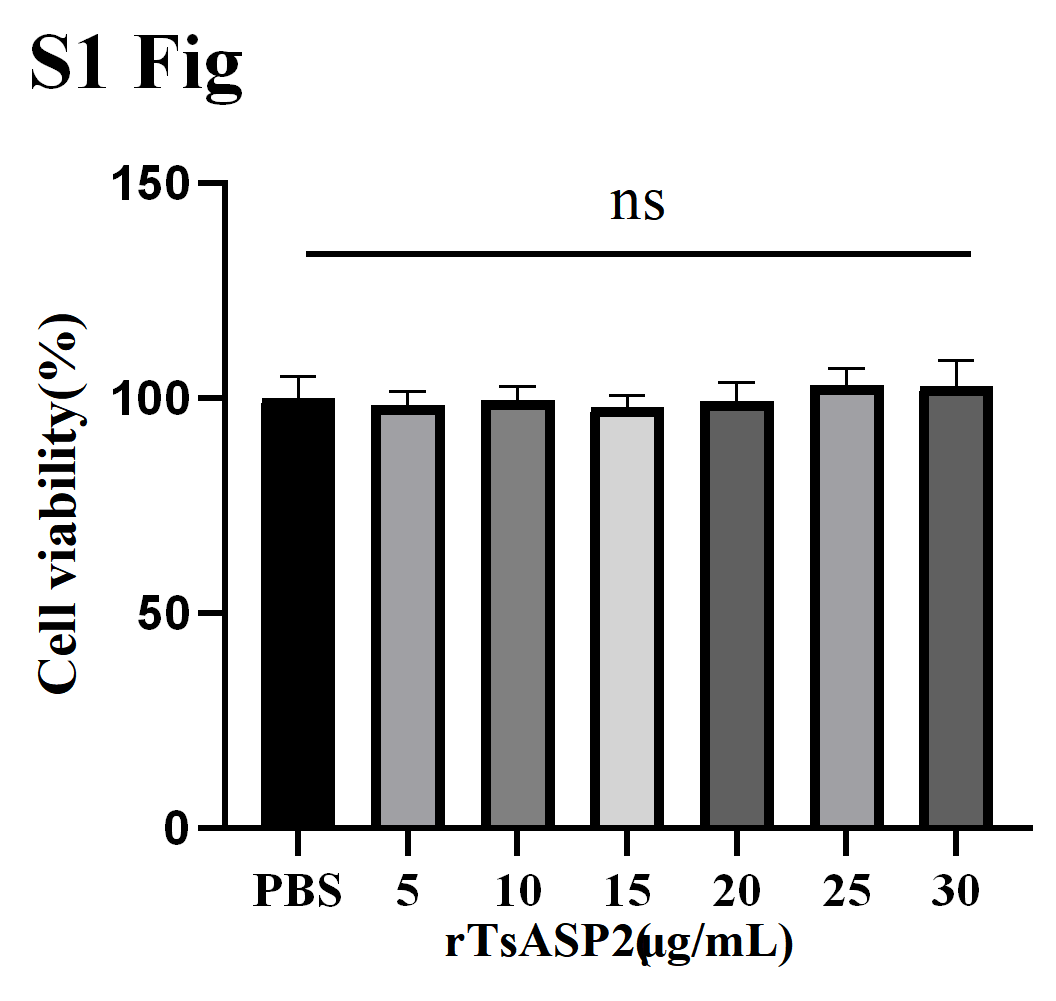
**

**S1 Fig. CCK-8 assay for the viability of Caco-2 cells treated with rTsASP2**

Effect of various concentrations of rTsASP2 on Caco-2 cell viability; the data shown are means ± SD. Representative results from one out of two independent experiments with n = 5. ns: no significant difference
