# Supplementary material for: Aspartic protease 2 from Trichinella spiralis excretion/secretion products hydrolyzes tight junctions of intestinal epithelial cells
Source: PLoS Negl Trop Dis. 2025 Dec 8;19(12):e0013805. doi: 10.1371/journal.pntd.0013805 (PMC12700411; doi:10.1371/journal.pntd.0013805)
Supplement: S2 Fig — α. (DOCX) [file pntd.0013805.s005.docx]

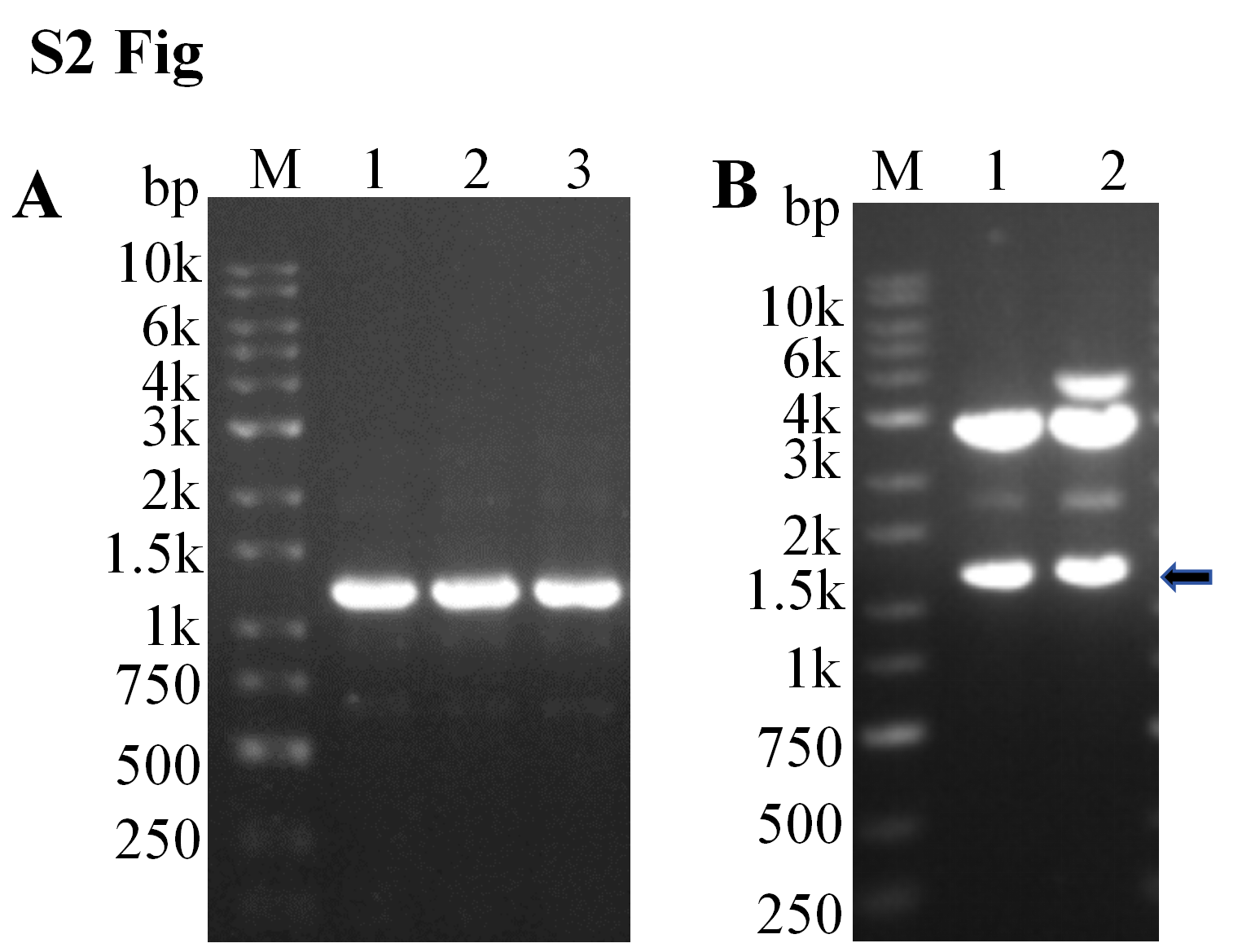


**S2 Fig. Verification of the recombinant pMD19-T/*TsASP2*/DH5α**

(**A**) PCR verification of recombinant pMD19-T/*TsASP2*/DH5α. M: DNA marker; 1-3: PCR product of *TsASP2*. (**B**) Restriction enzyme digestion verification of the plasmid pMD19-T/*TsASP2*. M: DNA marker; 1-2: Digested products of pMD19-T/*TsASP2* with *Nco*I and *Xba*I; the arrow indicated *TsASP2*.
