# Supplementary material for: Aspartic protease 2 from Trichinella spiralis excretion/secretion products hydrolyzes tight junctions of intestinal epithelial cells
Source: PLoS Negl Trop Dis. 2025 Dec 8;19(12):e0013805. doi: 10.1371/journal.pntd.0013805 (PMC12700411; doi:10.1371/journal.pntd.0013805)
Supplement: S3 Fig — (DOCX) [file pntd.0013805.s006.docx]

**
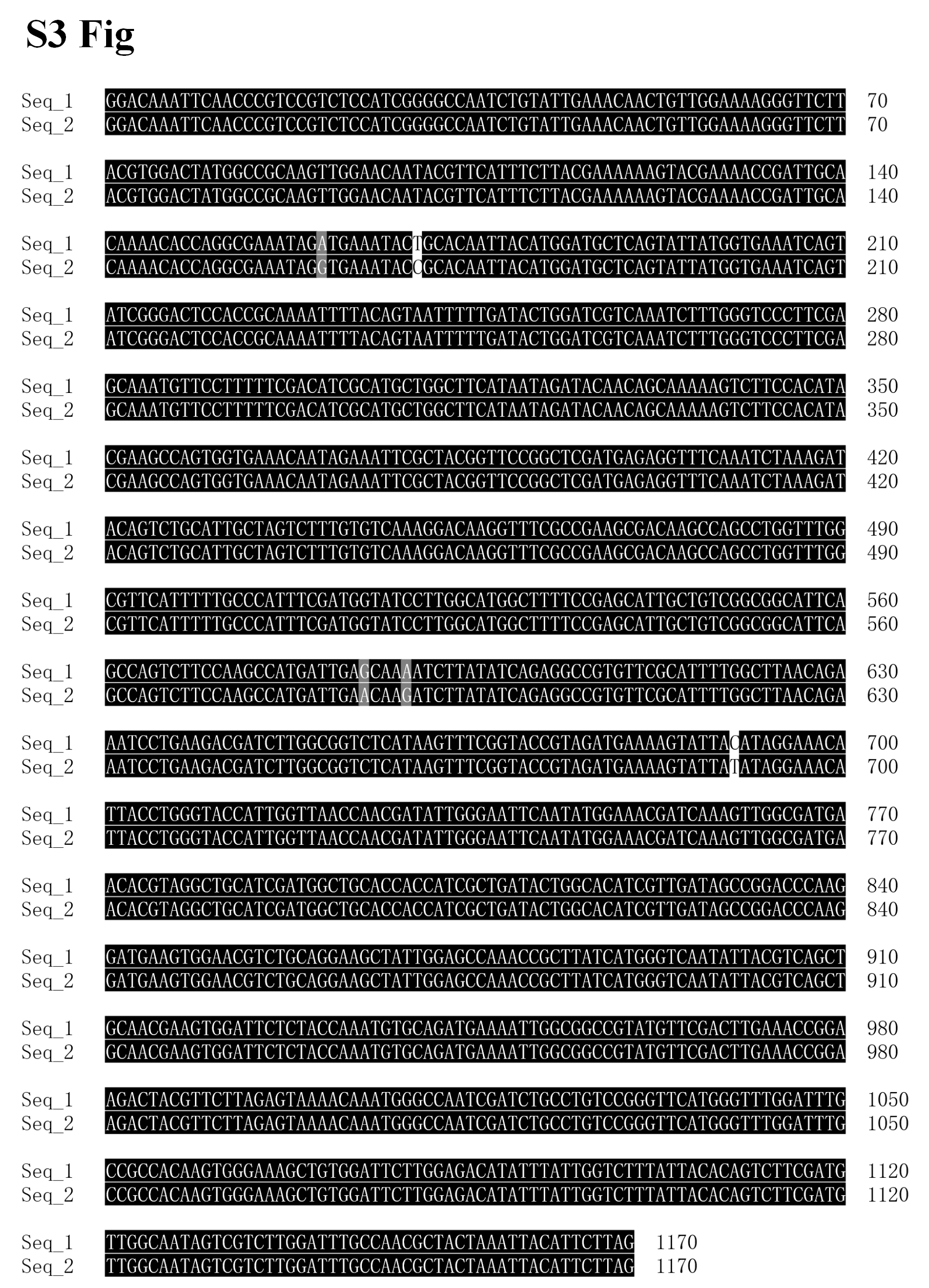
**

**S3 Fig. Comparison of recombination plasmid sequencing**

Seq_1: Sequence in GenBank for the *TsASP2* fragment; Seq_2: Sequencing result of target gene in pMD19-T/*TsASP2*/DH5α
