# Supplementary material for: Aspartic protease 2 from Trichinella spiralis excretion/secretion products hydrolyzes tight junctions of intestinal epithelial cells
Source: PLoS Negl Trop Dis. 2025 Dec 8;19(12):e0013805. doi: 10.1371/journal.pntd.0013805 (PMC12700411; doi:10.1371/journal.pntd.0013805)
Supplement: S4 Fig — (DOCX) [file pntd.0013805.s007.docx]

**
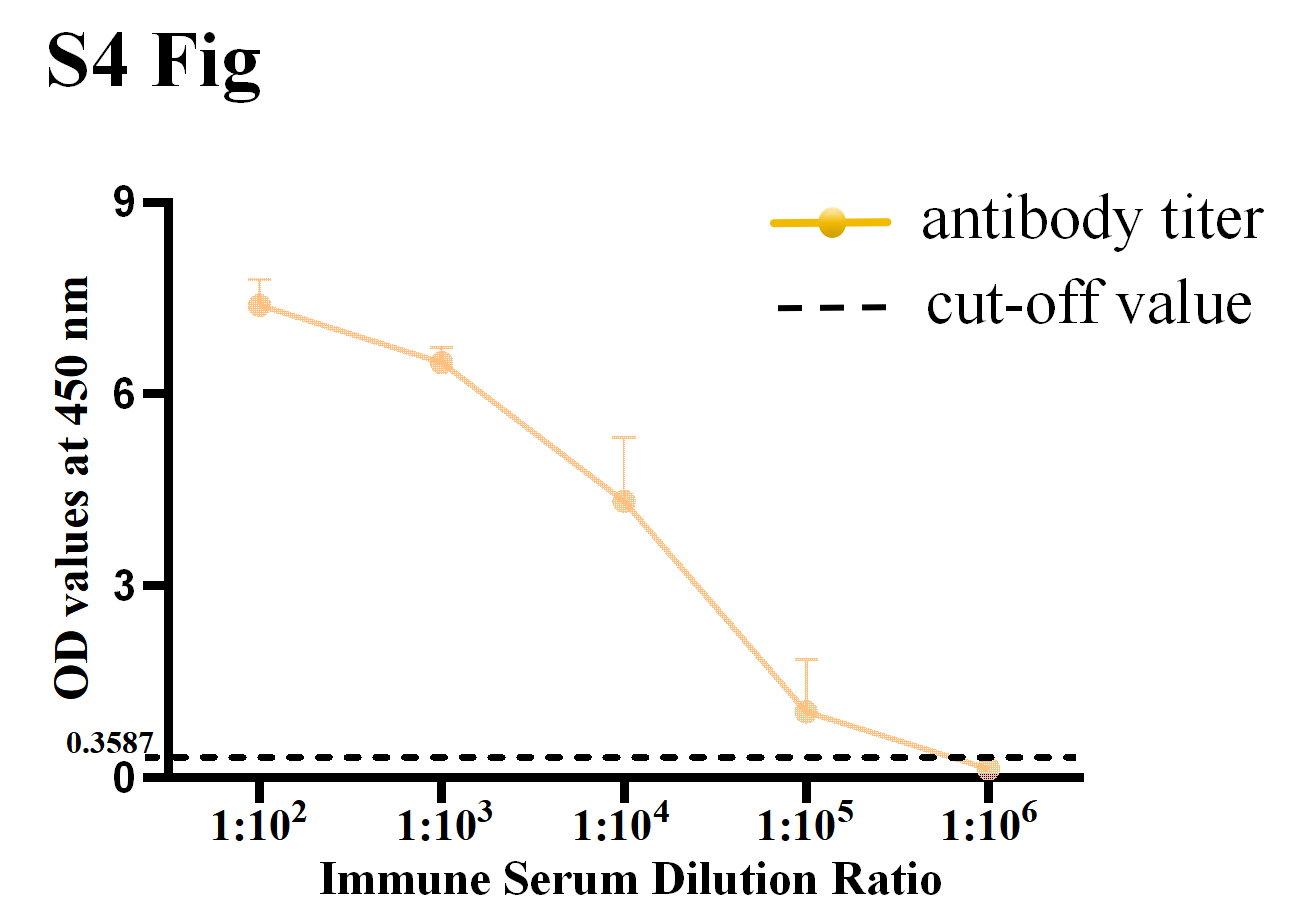
**

**S4 Fig. Serum antibody titer in rTsASP2-immunized mice**

The data shown are means ± SD, with n=3 for antibody titer measurement and n=17 for the determination of the cut-off value.
