# Supplementary material for: Aspartic protease 2 from Trichinella spiralis excretion/secretion products hydrolyzes tight junctions of intestinal epithelial cells
Source: PLoS Negl Trop Dis. 2025 Dec 8;19(12):e0013805. doi: 10.1371/journal.pntd.0013805 (PMC12700411; doi:10.1371/journal.pntd.0013805)
Supplement: S5 Fig — (DOCX) [file pntd.0013805.s008.docx]

**
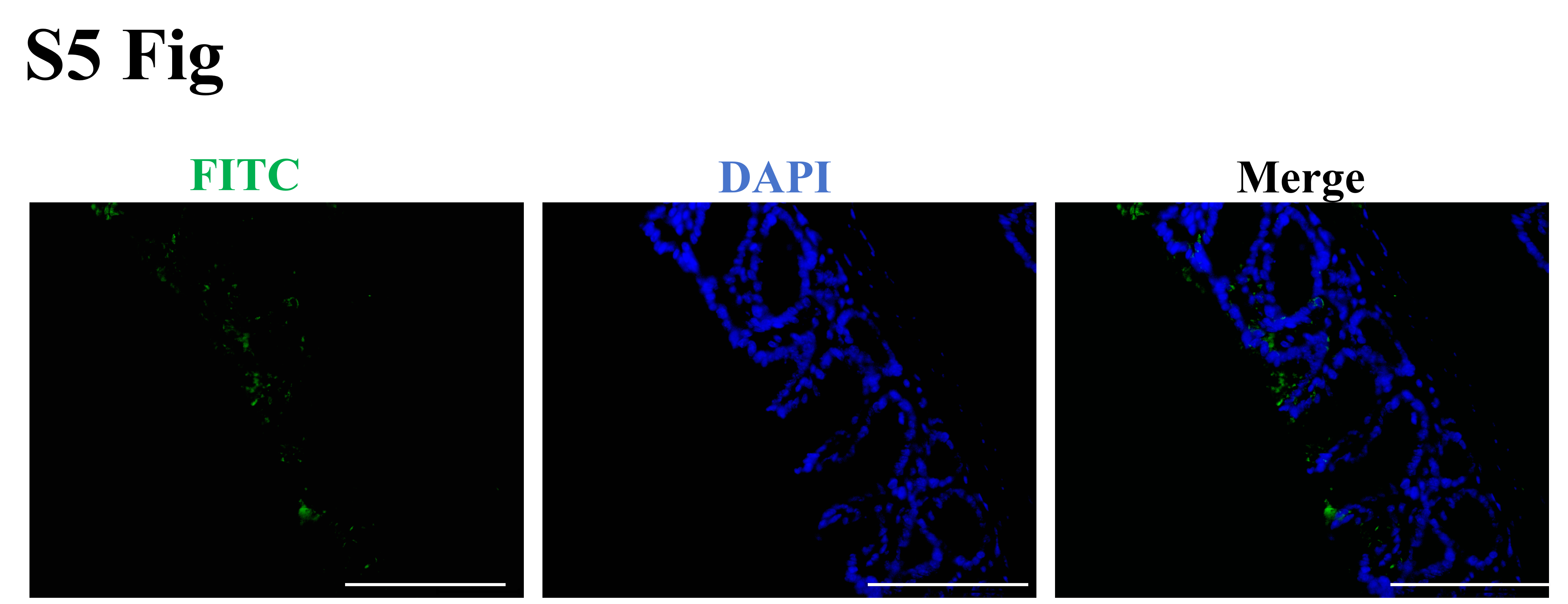
**

**S5 Fig. The recombinant *L. lactis* expressing rTsASP2 in the colonic epithelium**

Positive green fluorescence staining was observed in the colonic epithelium of mice immunized with recombinant *L. lactis* at day 5 after immunization. The nuclei of intestinal cells were stained blue by DAPI. Scale bar = 100 μm.
